# Effect of Fermented Red Ginseng Concentrate Intake on Stool Characteristic, Biochemical Parameters, and Gut Microbiota in Elderly Korean Women

**DOI:** 10.3390/nu14091693

**Published:** 2022-04-19

**Authors:** Songhee Lee, Sunghee Jung, Heesang You, Yeongju Lee, Youngsook Park, Hyunkoo Lee, Sunghee Hyun

**Affiliations:** 1 Department of Biomedical Laboratory Science, Graduate School, Eulji University, Uijeongbu-si 11759, Korea; song-1107@naver.com (S.L.); dlddwn1@gmail.com (Y.L.); 2 Department of Internal Medicine, College of Medicine, Eulji University, Daejeon-si 35233, Korea; jsh@eulji.ac.kr; 3 Department of Senior Healthcare, Graduate School, Eulji University, Uijeongbu-si 11759, Korea; yhs1532@nate.com; 4 Department of Gastroenterology, Nowon Eulji University Hospital, Eulji University School of Medicine, Seoul 01830, Korea; pys1109@eulji.ac.kr; 5 LHK Fermentation Lab., Seongnam-si 13209, Korea; hgnine@naver.com

**Keywords:** bowel movements, elderly individual, fermented red ginseng concentrate, glucose, gut microbiota

## Abstract

Fermented red ginseng (FRG) has been used as a general stimulant and herbal medicine for health promotion in Asia for thousands of years. Few studies have investigated the effects of FRG containing prebiotics on the gut microbiota. Here, 29 Korean women aged ≥ 50 years were administered FRG for three weeks to determine its effect on stool characteristics, biochemical parameters, and gut microbiome. Gut microbial DNA was subjected to 16S rRNA V3–V4 region sequencing to assess microbial distribution in different stages. Additionally, the stool consistency, frequency of bowel movements, and biochemical parameters of blood were evaluated. We found that FRG intake improved stool consistency and increased the frequency of bowel movements compared to before intake. Biochemical parameters such as glucose, triglyceride, cholesterol, low-density lipoprotein cholesterol, creatinine, alkaline phosphatase, and lactate dehydrogenase decreased and high-density lipoprotein cholesterol increased with FRG intake. Gut microbiome analysis revealed 20 specific bacteria after three weeks of FRG intake. Additionally, 16 pathways correlated with the 20 specific bacteria were enhanced after red ginseng intake. In conclusion, FRG promoted health in elderly women by lowering blood glucose levels and improving bowel movement frequency. The increase in bacteria observed with FRG ingestion supports these findings.

## 1. Introduction

Ginseng is a perennial herb that has been used in traditional medicine for thousands of years in various Asian countries [1,2]. Ginseng refers to the root of several species of the genus *Panax* (CA Meyer Araliaceae) and is generally harvested and dried after 3 years of growth [2]. Red ginseng is steamed, and dried ginseng and the content of ginseng saponin called ginsenoside is high [3]. Growing evidence suggests that red ginseng is potentially effective for alleviating obesity, diabetes, and dyslipidemia [4]. And red ginseng is reportedly associated with metabolism, human diseases, organismal systems, environmental information processing, and cellular processes [5]. Interestingly, fermented red ginseng (FRG) with fermented and enzymatically treated red ginseng exhibits higher and faster absorption of ginsenosides than unfermented red ginseng because it is richer in compound K among ginsenosides [5,6].

After oral administration, ginseng and red ginseng are usually exposed to gastric acid or digestive and bacterial enzymes. Before absorption, ginsenosides are metabolized by the intestinal microflora and absorbed into the intestine and blood. The gut microbiota plays an important role in the gastrointestinal tract and is associated with overall human health and metabolism [7]. There are approximately 10^14^ bacterial cells in the human gastrointestinal tract, which is 10 times the total number of somatic cells [8]. The gut microbiome influences nutrient absorption, energy use, storage, immune system, and metabolic disease pathogenesis [9]. The mechanism by which the gut microbiota improves health is not fully understood, but it is influenced by the diet; the intake of agents such as prebiotics contributes to its environmental composition [10]. Although FRG and its components correlate with changes in the host gut microbiota, the detailed molecular mechanisms underlying their beneficial effects have not been fully elucidated. Most studies have evaluated the effect of a single formulation of red ginseng on intestinal bacteria in animals and human patients [11].

Understanding how the human gut microbiota influences the aging process is challenging [12]. The gut is composed of a complex ecosystem of organisms that varies over time, with differences in composition in the elderly and young populations [13]. Elderly people show a physiological decline in several biological functions, including gastrointestinal dysfunction. With age, the production of saliva, perception of taste, and function of chewing deteriorate; furthermore, the secretion of gastric acids and passage through the gastrointestinal tract decrease, leading to constipation [14]. Additionally, the overall abundance of the gut microbiome decreases, whereas the abundance of certain groups of bacteria or bacteria associated with senescence increases.

The normal gut microbiome relies on host nutrient signaling pathways to exert beneficial effects on host health and longevity and communicates with the host through a variety of mechanisms [15]. There are considerable interindividual variations in the gut microbiota, and in particular, the gut microbiota of men and women differs depending on various factors, including sex hormones [16]. In addition, there may be intra-individual variations in the gut microbiome, particularly among women of different ages; the different gut microbiome structures may be due to menstrual-associated estrogen [17]. To determine the effect of FRG intake in a specific group, in this study, we limited the subjects to postmenopausal women. A study on the intake of fermentation-related preparations such as prebiotics showed that changes in the intestinal microflora of healthy adults after food supplementation can occur even in a short period of one week [18]. It has been reported that intestinal microflora returns to the previous level if the supplementation is stopped [19]. Therefore, in this study, the intake of probiotics previously taken was stopped for three weeks before starting the study. We aimed to evaluate the effect of a three-week intake of FRG concentrating on the gut microbiome, stool characteristics, and biochemical parameters in elderly Korean women.

## 2. Materials and Methods

### 2.1. Fermentation of Red Ginseng

FRG was manufactured by LHK Fermentation Lab (LHK Fermentation Lab., Seongnam-si, Korea). Briefly, red ginseng was fermented by inoculating 1 billion cells (0.1–0.5% *v*/*v*) of both *Lactobacillus plantarum* and *L. brevis* in 25% diluted Korean red ginseng solution. The resulting mixture was incubated until the bacterial growth reached 10^6^–10^7^/g. This mixture was used as the starter culture. The nutritional content and ginsenoside analysis results of FRG are shown in Table S1 and Figure S1, respectively.

### 2.2. Study Design

**Figure 1:**
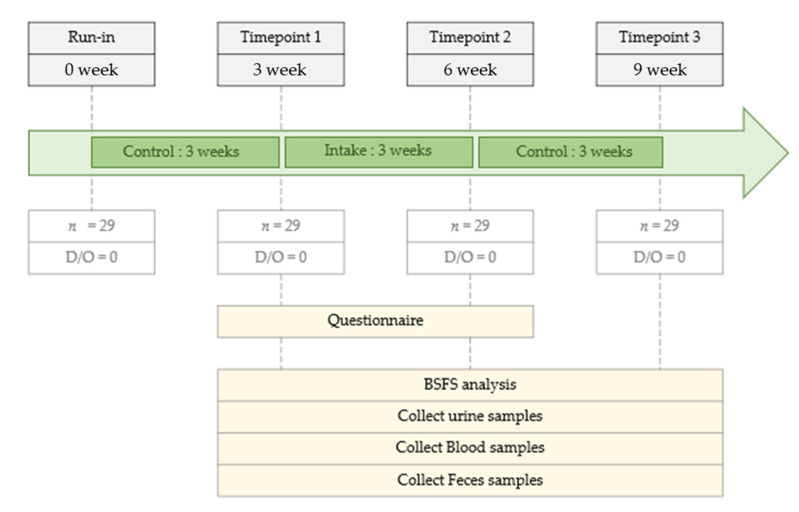
Study design.

This was a longitudinal pilot study initiated on July 1, 2019, and controlled using a time series over 9 weeks (Figure 1). The study was divided into the following three time points: 3, 6, and 9 weeks. The first 3 weeks (0 to 3) were the run-in period, wherein all participants maintained their usual dietary habits. At time point 1 (TP1, week 3), the participants were administered commercial FRG (20 mL) daily, 30 min before meals, for 3 weeks until time point 2 (TP2). The control period was from TP2 to time point 3 (TP3, weeks 6–9), when all participants maintained their usual dietary habits. During the 3-week intake period of the total 9-week study period, the participants consumed only FRG provided by us. The study protocol was approved by the Internal Review Board (IRB) of Eulji University (IRB No. EUIRB 2019-53).

### 2.3. Participants

Twenty-nine healthy female volunteers from the Miraeseum Seongnam Senior Complex in Seongnam City, Gyeonggi-do Province, Korea, were included in the study. Participants in this study submitted the Informed Consent Form. The participants were healthy, postmenopausal women aged ≥ 50 years (Table 1). The exclusion criteria are as follows. (1) Individuals taking drugs that may affect metabolic and vascular functions, such as diabetes medications and blood pressure-lowering drugs. (2) Patients with uncontrolled hypertension (systolic blood pressure ≥ 160 mmHg or diastolic blood pressure ≥ 100 mmHg), high fasting blood glucose level (≥126 mg/dL), diabetes, a history of cardiovascular disease, autoimmune disease, thyroid disease, or cancer. (3) Those who have already participated in another study. Participants were instructed to maintain their usual lifestyle during the study period and report adverse events. A questionnaire was distributed at each visit, and intake compliance was monitored once a week via a phone interview. The participants were instructed to immediately contact the researchers if they experienced any adverse reactions.

### 2.4. Participant Details and Sample Collection

All participants completed a survey of their family medical history, antibiotic use, bowel movement, and diet (data not shown). We measured the height and weight of the participants barefoot and in light clothing, respectively. Waist circumference was measured just above the hipbone with the tape leveled. Body mass index (BMI) was calculated by dividing body weight (kg) by the square of height (m^2^), according to the WHO 2000 guidelines [20,21]. Blood pressure was evaluated after the participants had rested for more than 5 min while seated.

Blood samples were collected from the participants on an empty stomach. Venipuncture was performed using a vacuum collection tube, and the blood was stored in a blood collection tube, EDTA Vacutainer, and serum separator tube (Becton Dickinson, Franklin Lakes, NJ, USA). Blood samples were centrifuged at 1500× *g* for 15 min at 4 °C. Standard laboratory methods and certified biochemical and hematological tests were performed using an automated analyzer (Roche Diagnostics, Mannheim, Germany).

Urinalysis was performed using a urine reagent strip ComboStic 10 (DFIcare Co., Gimhae-si, Korea) and immersing the strip in a urine sample. The Laboratory technicians performed the test within 10 min after collecting the samples according to the manufacturer’s instructions, and the results were read within 5 min and the samples were discarded [22].

The day before each visit, all participants were provided a sterile stool container and instructed of their use. The participants were instructed to present at least 10 g of fresh stool. The night before the visit day or the morning of the visit day, the participants collected stool and stored it in a home refrigerator at 4 °C until submission. After receiving the sample, we immediately stored it in a 4 °C refrigerator and moved the sample to the laboratory within 2 h, where the DNA was immediately extracted from the samples [23].

### 2.5. Stool Characteristic

We surveyed the stool characteristic for the stool consistency and frequency of bowel movements, respectively. The stool consistency was evaluated by classification using the Bristol Stool Form Scale (BSFS) [24]. The BSFS is an ordinal scale of edge types 1 to 7, with 1 being the most difficult variable. Types 1 and 2 are considered unusually hard stools, and types 6 and 7 are considered unusually thin liquid stools. Types 3, 4, and 5 are generally considered the most “normal” form of stool, and type 4 is the baseline for cross-sectional studies of healthy adults [25]. The frequency of bowel movements was evaluated by examining the average frequency of defecation per week. Normal defecation frequency is usually defined as ≥3 times/week and ≤3 times/day [26]. Less than 3 times/week is classified as constipation, and more than 3 times/day is classified as diarrhea, indicating abnormal bowel movements [27].

### 2.6. Microbial DNA Extraction and 16S rRNA Amplicon Library Preparation and Sequencing

DNA extraction was performed using the QIAamp PowerFecal Pro-DNA Kit (Qi-agen, Hilden, Germany), following the manufacturer’s instructions. 16S rRNA amplicon library preparation and sequencing were performed using the Ion Library Fabrication Kit on an Ion Torrent S5xl platform (Thermo Fisher Scientific Inc., Waltham, MA, USA). The extracted DNA was subjected to PCR according to the protocol using the following universal primers, adapter sequences, and index sequences for the V3-V4 region of the bacterial 16S rRNA gene using a Platinum PCR SuperMix High Fidelity system (Thermo Fisher Scientific): 341F (5′-CCTACGGGNGGCWGCAG-3′), sample-specific 6–8-bp tag sequence, and 805R (5′-GACTACHVGGGTATCTAATCC-3′), respectively. The final volume was 27 μL, the template DNA was set at 2.5 ng, and the up and down primers were set at 50 nM. The thermal cycling conditions consisted of an initial denaturation at 94 °C for 3 min, followed by 30 cycles of 94 °C for 30 s, 50 °C for 30 s, and 70 °C for 30 s. Amplicon libraries were re-purified to remove residual primer dimers and contaminants using the Agencourt AMPure XP DNA Purification Kit (Beckman Coulter, Brea, CA, USA) according to the manufacturer’s instructions. Final eluted samples were eluted in 15 µL of low-EDTA Tris EDTA buffer [18]. DNA concentration and quality and amplicon library concentration were quantified using the Qubit dsDNA HS Assay Kit on a Qubit 4 fluorometer (Thermo Fisher Scientific). An Agilent 2100 Bioanalyzer system (Agilent Technologies, Palo Alto, CA, USA) was used to evaluate primer-dimer or detectable adapter contamination and fragment size of the pooled DNA [18]. The concentrated particles were loaded into an Ion 530 Chip Kit (Thermo Fisher Scientific), and sequencing was performed on an Ion GeneStudio S5 system (Thermo Fisher Scientific), following the manufacturer’s instructions [28,29,30]. High-throughput 16S rRNA gene amplicon sequencing was then conducted using S5, the next-generation sequencer on the Ion Torrent S5XL platform.

### 2.7. Analysis of 16S rRNA Amplicon Sequences

The FASTQ file containing the raw data of 16S rRNA sequences was obtained using Torrent Suite Software version 5.14.1.1. (Thermo Fisher Scientific Inc., Waltham, MA, USA). The 16S rRNA workflow module in EzBioCloud software (ChunLab, Seoul, Korea) [31] was used to classify individual reads by combining the Basic Local Alignment Search Tool with the curated Greengenes Database, which contains a high-quality library of full-length 16S rRNA sequences.

Reads shorter than 500 bp or inappropriately paired were excluded from the analysis. In addition, chimeras were removed from the sequence data. Sequences were clustered into operational taxonomic units at 97% identity using QIIME pick_open_reference_otus.py, the Greengenes 13.5 reference database, and the UCLUST algorithm.

### 2.8. Statistical Analysis

Significance and mean analyses were performed using IBM SPSS version 20.0 (SPSS, Chicago, IL, USA). Line graphs, scatter plots, and heat maps were generated using GraphPad Prism software version 8.3.1 (GraphPad Software, San Diego, CA, USA). For the heat map, correlation analysis was performed using Pearson’s correlation coefficient, and the symbol was marked with “*r*”. Parametric pairwise *t*-tests were used to compare two different time points (TP) and determined and calculated the mean and standard deviation of the dependent variable. Significance was set at *p* < 0.05. Linear discriminant effect size (LEfSe) analysis was performed to identify biomarkers between two or among more groups based on their relative abundance using the Galaxy Hutlab online platform [32,33]. Feature and histogram analysis of one plot identified biomarkers based on specific genera and species in different sample groups. For metabolic profiling, the Kyoto Encyclopedia of Genes and Genomes (KEGG) database was used as a reference for genes related to microbial taxa. And pathways were analyzed using Phylogenetic Investigation of Communities by Reconstruction of Unobserved States (PICRUSt) [34]. The KEGG pathway predicted based on the estimated genomic content of the gut microbiota was categorized into functional pathways such as metabolism, human diseases, organismal systems, environmental information processing, and cellular processes.

## 3. Results

### 3.1. Participant Information

A total of 40 participants were screened, of which 29 met the inclusion criteria, were enrolled in the study, and completed the study (Figure 1). Participants’ basic information is summarized in Table 1. The age category of the participants was 50 years and older, all female, with a BMI of 18–25 kg/m^2^ and normotensive blood pressure (systolic < 130 mmHg and diastolic < 80 mmHg). None of the participants took FRG before participating in this study. In addition, no adverse effects of FRG were observed in the participants during the study.

### 3.2. Stool Characteristic

**Figure 2:**
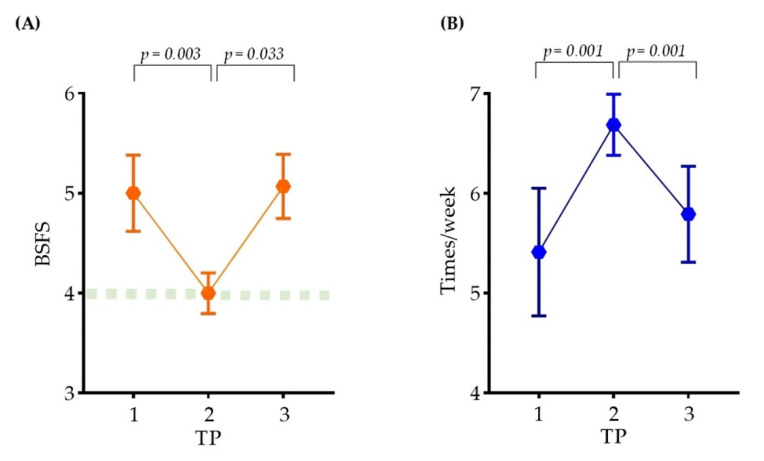
Changes in bowel movements in participants. A line graph showing the change in the (**A**) stool consistency and (**B**) participants’ frequency of bowel movements at each time point. (**A**) Stool consistency was indicated using BSFS. (**B**) Participants’ frequency of bowel movements were presented at 1 week. The points of the graph represent the mean, the connecting line represents the slope, and the bar represents the standard error of the mean (SEM). SEM was obtained by taking standard deviation (SD) and dividing by the square root of the sample size. For statistics, paired t-test was performed.

At each time point (TP1, TP2, and TP3), the stool consistency and frequency of bowel movements were investigated (Figure 2). The stool consistency samples were evaluated by classification using the Bristol Stool Form Scale (BSFS). According to the BSFS, the feces were classified as type 5 in TP1, type 4 in TP2, and type 5.07 in TP3. The frequency of bowel movements was evaluated by examining the average frequency of defecation per week. The mean bowel movements were 5.41 in TP1, 6.68 in TP2, and 5.79 in TP3.

### 3.3. Changes in the Biochemical Parameters after FRG Intake

We performed blood and urine tests to determine the changes that occurred after FRG intake. FRG did not induce any adverse effects, such as an abnormal range of white blood cells and blood in the urine (Tables S2–S6). We observed changes in the biochemical parameters of participants after FRG intake (Table 2). The mean fasting blood glucose level was 87.0 mg/dL at TP1 and 80.7 mg/dL at TP2, decreasing by 9.2% at TP2 (*p* = 0.000). Three weeks after stopping FRG intake, the fasting blood glucose level returned to 85.3 mg/dL, similar to that before FRG intake (*p* = 0.000). The alkaline phosphatase (ALP) level decreased from 77.6 at TP1 to 72.1 at TP2 (*p* = 0.000), and increased to 74.9 at TP3 (*p* = 0.002). The lactate dehydrogenase (LDH) content decreased from 187.9 at TP1 to 177.6 at TP2, and it was almost constant until TP3. The triglyceride (TG) level decreased sharply from 200.7 at TP1 to 155.4 at TP2. However, it decreased to 149 at TP3 and was not significant at any time point. The low-density lipoprotein cholesterol (LDL) level decreased from 129.6 at TP1 to 115.4 at TP2 and increased to 125.7 at TP3.

### 3.4. Phylogenetic Analysis of the Gut Microbiota

**Figure 3:**
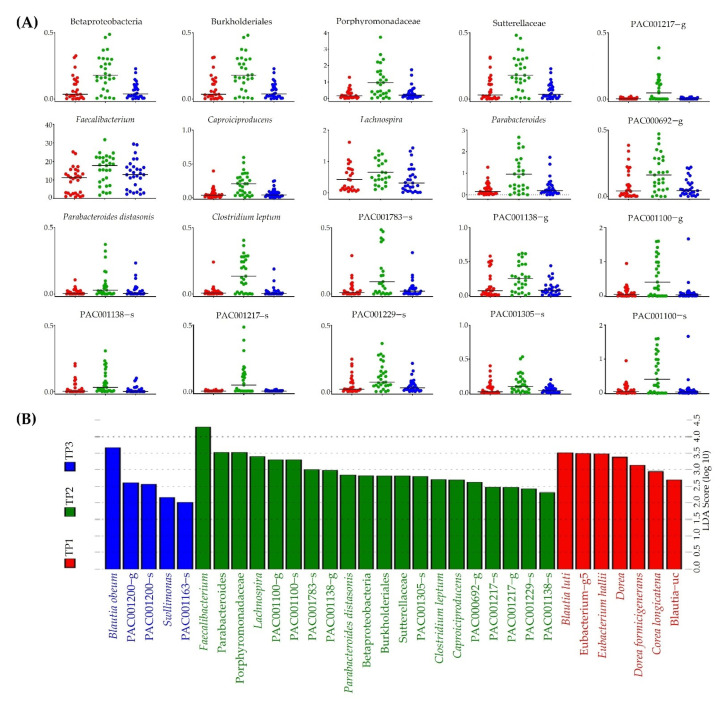
Observation of significantly enriched bacteria at each time point following FRG intake. (**A**) Relative abundance by time points of 20 specific strains observed in TP2. (**B**) Twenty specific strains significantly observed in TP2 (green) compared to TP1 (red) and TP3 (blue).

We used LEfSe to identify taxa that exhibited biological differences at the bacterial genus or species level at each time point (Figure 3). Four bacterial taxa (*Blautia obeum*, PAC001200_g, PAC001200_s, and *Sellimonas*) were relatively abundant at TP1. Meanwhile, 20 bacteria (*Faecalibacterium*, *Parabaceroides*, Porphyromonadaceae sp., *Lachnospira*, PAC001100_g, PAC001100_s, PAC001783_s, PAC001138_g, *Parabacteroides distasonis*, *Betaproteobacteria* sp., Burkholderiales sp., Sutterellaceae sp., PAC001305_s, *Clostridium leptum*, *Caproiciproducens*, PAC000692_g, PAC001217_s, PAC001217_g, PAC001229_s, and PAC001138_s) were relatively abundant at TP2. Seven bacteria (*Blautia luti*, *Eubacterim*_g5, *Eubacterium hallii*, *Dorea* sp., *Dorea formicgenerans*, *Dorea longicatena*, and *Blautia*_uc) were relatively abundant at TP3. We observed an abundance of 20 bacteria at TP2 (Figure 3A).

### 3.5. Correlation between Important Indicators and the Gut Microbiota

We observed significant changes in the stool consistency, frequency of bowel movements, and biochemical parameters after the intake of FRG (TP2) (Figure 2 and Table 2). In addition, we observed 20 specific bacteria that were significantly enriched after the intake of FRG (TP2) (Figure 3).

**Figure 4:**
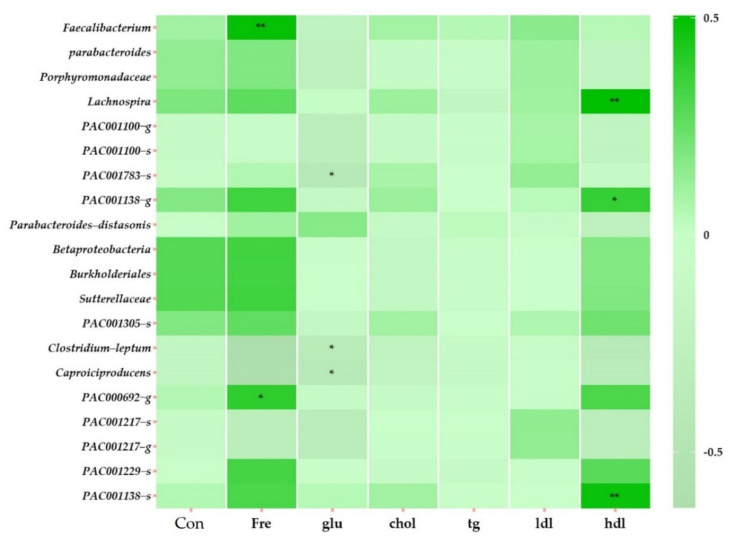
Heatmap clustering showing correlations with variables in TP2 of all subjects. The displayed heatmap clustering at the taxonomy level is genus and species. The distance measurement was performed with Pearson, and the clustering algorithm was performed with Average. The clustering algorithm refers to measuring the distance between each cluster, and when measuring the distance between two clusters, the average connection was performed by calculating the average by calculating the distance between all points in the cluster. Abbreviations: Con, stool consistency; Fre, frequency of bowel movements; glu, glucose; chol, cholesterol; tg, triglycerides; ldl, low-density lipoprotein cholesterol; hdl, high-density lipoprotein cholesterol. * *p* > 0.05. ** *p* > 0.01.

We selected significant variables among the variables observed at TP2 (stool consistency, frequency of bowel movements, and biochemical parameters), and a heatmap clustering was performed to determine correlations among the 20 specific bacteria (Figure 4). The frequency of bowel movements correlated with *Faecalibacterium* (*r =* 0.50, *p =* 0.007), *Clostridium leptum* (*r =* −0.629, *p =* 0.000), *Caproiciproducens* (*r =* −0.611, *p =* 0.000), and PAC000692_g (*r =* 0.386, *p =* 0.039). The glucose level negatively correlated with PAC001783_s (*r =* −0.428, *p =* 0.020), *C. leptum* (*r =* −0.371, *p =* 0.047), and *Caproiciproducens* (*r =* −0.408, *p =* 0.028). The HDL level positively correlated with *Lachnospira* (*r =* 0.506, *p* = 0.005), PAC001138_g (*r =* 0.371, *p =* 0.048), and PAC001138_s (*r =* 0.477, *p =* 0.009).

### 3.6. Predictive Pathway Analysis Based on Bacterial Functions

**Figure 5:**
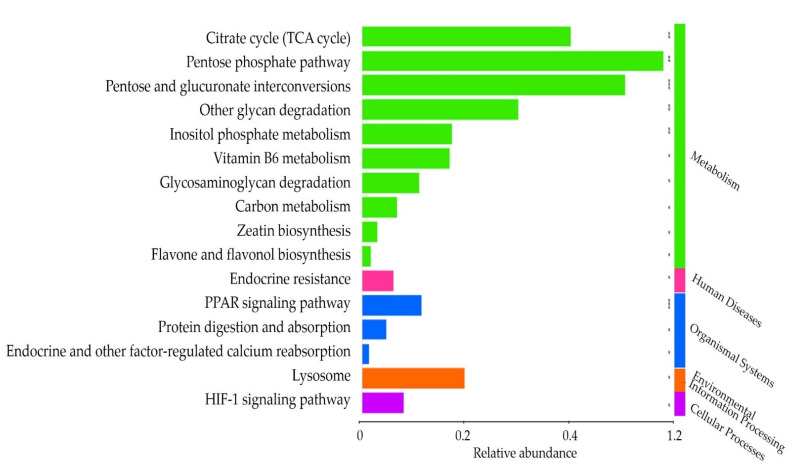
Significantly observed pathways in TP2. Based on the KEGG database, we used PICRUSt to predict the module and MinPath to predict the path. * *p* > 0.05. ** *p* > 0.01. *** *p* > 0.005.

Based on the genomic database, we targeted the gut microbiota after the intake of FRG (TP2). Gene abundance was analyzed based on the results of 16S taxonomic profiling information (Figure 5). We estimated gene abundance and selected 16 pathways that were observed to be significantly enriched at TP2 (cutoff 1.0%).

Of the 16 pathways, 10 were classified as a metabolism network, one as a human diseases network, three as an organismal systems network, one as an environmental information processing network, and one as a cellular processes network. The metabolism categories included citrate cycle (TCA cycle), pentose phosphate pathway, pentose and glucuronate interconversions, other glycan degradation, inositol phosphate metabolism, vitamin B6 metabolism, glycosaminoglycan degradation, carbon metabolism, zeatin biosynthesis, and flavone and flavonol biosynthesis. Endocrine resistance was classified into the category of human diseases. The organismal systems category comprised the PPAR signaling pathway, protein digestion and absorption, and endocrine and other factor-regulated calcium reabsorption. Lysosome was classified in the category of environmental information processing. The HIF-1 signaling pathway was classified in the cellular processes category.

### 3.7. Correlation between the Significant Pathways and the Gut Microbiota

**Figure 6:**
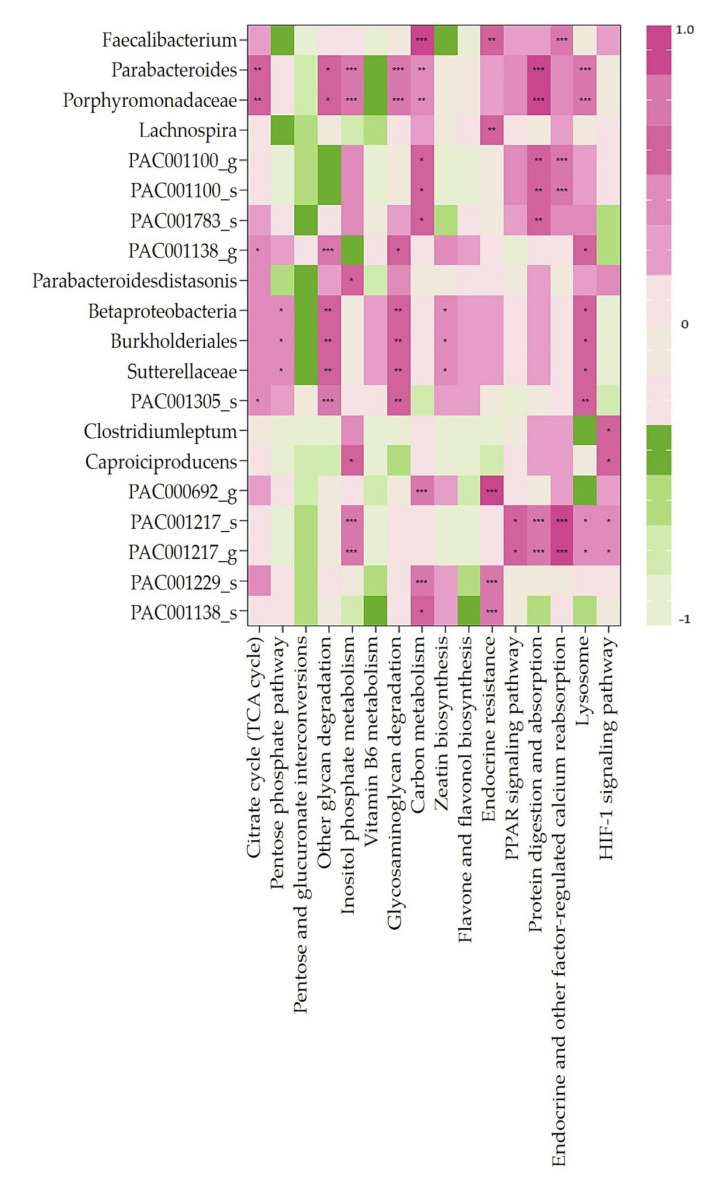
Heatmap clustering showing correlation analysis between significantly observed pathways and 20 bacteria enriched only in TP2. Spearman correlation analysis between predictive pathways and specific bacteria of interest. * *p* > 0.05. ** *p* > 0.01. *** *p* > 0.005.

We analyzed the metabolic pathways related to all strains detected at TP2 (Figure 5). We further analyzed the correlation between the 20 bacteria that were significantly enriched after FRG intake (TP2) and 16 pathways significantly involved (Figure 6).

The citrate cycle (TCA cycle) was correlated with 4 of the 20 specific bacteria (*Parabacteroides* sp., *Porphyromonadaceae* sp., PAC001138_g, and PAC001305_s). The pentose phosphate pathway was correlated with three bacteria (*Betaproteobacteria* sp., *Burkholderiales* sp., and *Sutterellaceae* sp.). The other glycan degradation pathways were correlated with seven bacteria (*Parabacteroides* sp., *Porphyromonadaceae* sp., PAC001138_g, *Betaproteobacteria* sp., *Burkholderiales* sp., *Sutterellaceae* sp., and PAC001305_s). The inositol phosphate metabolism pathway was correlated with six bacteria (*Parabacteroides* sp., *Porphyromonadaceae* sp., *Parabacteroidesdistasonis* sp., *Caproiciproducens*, PAC001217_s, and PAC001217_g). The glycosaminoglycan degradation pathway was correlated with seven bacteria (*Parabacteroides* sp., *Porphyromonadaceae* sp., PAC001138_g, *Betaproteobacteria* sp., *Burkholderiales* sp., *Sutterellaceae* sp., and PAC001305_s). The carbon metabolism pathway was correlated with nine bacteria (*Faecalibacterium*, *Parabacteroides* sp., *Porphyromonadaceae* sp., PAC001100_g, PAC001100_s, PAC001783_s, PAC000692_g, PAC001229_s, and PAC001138_s). The zeatin biosynthesis pathway was correlated with three bacteria (*Betaproteobacteria* sp., *Burkholderiales* sp., and *Sutterellaceae* sp.). The endocrine resistance pathway involved five bacteria (*Faecalibacterium*, *Lachnospira*, PAC000692_g, PAC001229_s, and PAC001138_s). The PPAR signaling pathway was correlated with two bacteria (PAC001217_s and PAC001217_g). Protein digestion and absorption pathways were correlated with seven bacteria (*Parabacteroides* sp., *Porphyromadaceae* sp., PAC001100_g, PAC001100_s, PAC001783_s, PAC001217_s, and PAC001217_g). Endocrine and other factor-regulated calcium reabsorption pathways were correlated with five bacteria (*Faecalibacterium*, PAC001100_g, PAC001100_s, PAC001217_s, and PAC001217_g). The lysosome pathway was correlated with nine bacteria (*Parabacteroides* sp., *Porphyromonadaceae* sp., PAC001138_g, *Betaproteobacteria* sp., *Burkholderiales* sp., *Sutterellaceae* sp., PAC001305_s, PAC001217_s, and PAC001217_g). The HIF-1 signaling pathway was correlated with three bacteria (*C. leptum*, *Caproiciproducens*, PAC001217_s, and PAC001217_g).

## 4. Discussion

We found that FRG intake improved stool consistency and increased the frequency of bowel movements. Glucose, TG, cholesterol, LDL, creatinine, ALP, and LDH levels decreased and HDL levels increased with FRG intake. Moreover, gut microbiota was altered after three weeks of FRG ingestion and 20 specific bacteria were identified. In addition, 16 pathways enhanced by three weeks of FRG uptake were observed and correlated with 20 specific bacteria. Although there are studies on the effect of red ginseng on the intestinal microflora, there are no studies on the effect of FRG containing prebiotics on the gut microbiota [5]. With aging, defecation disorders and intestinal microbial imbalance appear, and it is difficult to identify signal transduction pathways and related mechanisms [12]. Therefore, in this study, changes in stool characteristics, biochemical parameters, intestinal microbiota, and pathways were observed in Korean women according to FRG intake for three weeks.

Assessment of stool consistency can elucidate normal or altered bowel habits [35]. Several stool morphology scales have been developed, and the most widely used scale is the BSFS [24]. The viscosity of stool is largely determined by the water content in the stool [36]. The BSFS is an ordinal scale of edge types 1 to 7, with 1 being the most difficult variable. Types 1 and 2 are considered unusually hard stools, and types 6 and 7 are considered unusually thin liquid stools. Types 3, 4, and 5 are generally considered the most “normal” form of stool, and type 4 is the baseline for cross-sectional studies of healthy adults. The stool consistency is exacerbated as the content of soluble solids increases due to a decrease in absorption of soluble dietary components such as carbohydrates [37]. In this study, the BSFS increased from 5 at TP1 to 4 at TP2. Although it was changed from 5 to 4, which is the normal range, the participants in this study significantly increased the abundance of carbohydrate metabolism after ingestion of FRG. This is explained by the lower fecal water content and increased water absorption in the gastrointestinal tract so that the water-absorbing small intestine can further aid in the breakdown of carbohydrates and fats [24]. Thus, the intake of FRG can regulate stool consistency.

Normal defecation frequency is usually defined as ≥3 times/week and ≤3 times/day [26]. Although the exact number of times has not been defined, it has been reported that the normal frequency varies from three times a week to three times a day. However, <3 times/week is classified as constipation and >3 times/day is classified as diarrhea, indicating abnormal bowel movements [27]. Defecation is related to changes in the intestinal microflora, and it may be affected by probiotics and dietary fiber and may vary slightly from one person to another [38]. Koreans consume the highest number of vegetables composed of dietary fiber in the world, and the active defecation of Koreans can be explained as follows [39]. Participants in this study answered that they defecated at a frequency of 5.41/weeks at TP1. The frequency of defecation increased to 6.68 at TP2, which is within the normal range. Various components, including galacto-oligosaccharides contained in FRG, are ingested, and when the dietary fiber intake is increased, stool characteristics may increase [40].

We observed 20 specific bacteria that were significantly enriched with FRG intake for three weeks. *Faecalibacterium* had a positive correlation with the frequency of bowel movements, carbon metabolism pathway, endocrine resistance pathway, and the endocrine and other factor-regulated calcium reabsorption pathways. *Fecalibacterium*, known to be correlated with rapid colonic transit, obtains its nutrients through the saccharification and fermentation of indigestible carbohydrates [41]. Additionally, this type of fermentation produces short-chain fatty acids (SCFAs), such as acetate and butyrate, which are major metabolites produced by the microorganisms in the large intestine through fermentation [42]. SCFAs are absorbed in exchange with bicarbonates, affecting the intestinal lumen pH and promoting microbial growth [43]. Although we did not measure SCFA in our study, a significant increase in *Faecalibacterium* abundance was observed. In addition, according to FRG intake, the carbon metabolism pathway was enriched, the frequency of bowel movements increased, and stool consistency was changed. Optimal intestinal calcium (Ca) absorption is required to protect bones and prevent osteoporosis [44]. In this study, the endocrine and other factor-regulated calcium reabsorption pathways were positively correlated with *Faecalibacterium* enriched with FRG intake. Given that calcium reabsorption metabolism is enriched, it is predicted that *Faecalibacterium* may be involved in intestinal calcium metabolism.

*C. leptum* and *Caproiciproducens* showed a negative correlation with glucose levels and a positive correlation with the HIF-1 signaling pathway. In addition, *Caproiciproducens* positively correlated with the inositol phosphate metabolic pathway. The HIF-1 signaling pathway and inositol phosphate metabolism are known to contribute to the intestinal barrier enhancing function [45,46]. Intestinal barrier dysfunction can lead to severe inflammatory responses due to the translocation of intestinal lumen-derived bacteria and endotoxins [47]. In this study, the participants increased the abundance of the intestinal barrier-enhancing functional pathway and significantly decreased LDH, a serum inflammatory marker, after ingestion of FRG. Therefore, it can be predicted that *C. leptum* and *Caproiciproducens* enriched by FRG contribute to intestinal barrier function. In a study of high-fat diet-induced metabolic disorders, an increased abundance of *Caproiciproducens* was observed in the group with improved blood lipid metabolism [48]. TG levels in participants in this study were 200.7 mg/dL at baseline TP1, which is borderline high (reference range 200–499 mg/dL). After three weeks of ingestion of FRG, TG levels decreased sharply to 155.4 mg/dL. This suggests that the intestinal microbial environment may have been improved due to the abundance of *Caproiciproducens*, an acid-producing bacterium that can utilize galactose as a carbon source [49]. *C. leptum*, which has been shown to improve insulin secretion in another study, increased abundantly with FRG intake in this study and may be involved in lowering glucose. It has been shown to affect glucose homeostasis by synergistically fermenting non-absorbable dietary carbohydrates, such as prebiotics, with other gut microbes. These results suggest that the *C. leptum* group could potentially enhance insulin secretion [50].

Porphyromonadaceae members and *Parabacteroides* observed in this study were positively correlated with the TCA cycle, other glycan degradation, inositol phosphate metabolism, glycosaminoglycan degradation, and carbon metabolism, protein digestion and absorption, and lysosomal pathways. *P. disstasonis* is positively correlated with inositol phosphate metabolism. The TCA cycle is responsible for the oxidative breakdown of sugars, fats, and amino acids, and plays a role in energy metabolism connecting various types of nutrients [51]. Other glycan degradation increases host nutrition by digesting glycans that cannot be broken down by the host, providing useful metabolites to the host [52]. Glycosaminoglycan Degradation Pathway Components Glycosaminoglycans (GAGs) are a major component of the animal’s extracellular matrix and are degraded by some bacteria, such as probiotics [53]. In a previous study, the abundance of *Parabacteroides* and *P. disstasonis* was reported to be relatively low in obese, inflammatory bowel disease (IBD) patients, and to be abundant in a group of patients with hyperglycemic improvement [54,55]. In this study, galacto-oligosaccharides, a prebiotic contained in FRG, promote the growth of beneficial bacteria in the gut. According to FRG intake, Porphyromonadaceae and *Parabacteroides* members were enriched, and they were more actively involved in energy and glycan breakdown metabolism. In addition, it is known that activation of the lysosomal pathway mediates intestinal protein absorption, and GAG is degraded in lysosomes containing hydrolases [56]. Protein digestion and absorption pathways, such as the lysosomal pathway, influence digestibility [57]. Thus, FRG uptake increases Porphyromonadaceae and *Parabacteroides* members and induces the promotion of metabolic and environmental information processes such as lysosomal pathways.

*Lachnospira* has been largely associated with the intake of a fiber-rich diet [58]. Its abundance has been reported to decrease in overweight and obese women [59]. In this study, *Lachnospira* showed a positive correlation with HDL, and to our knowledge, no study has observed the correlation between *Lachnospira* and HDL. In addition, *Lachnospira* showed a positive correlation with the endocrine resistance pathway. The endocrine resistance pathway is predicted to be a pathway related to the female reproductive system because all participants in this study were women.

Betaproteobacteria, Burkholderiales, and Sutterellaceae members showed correlations with the pentose phosphate pathway and other glycan degradation. They also showed a correlation between the GAG pathway and the lysosome pathway. It has been reported that the zeatin biosynthesis pathway correlates with sCD14, a bacterial translocation marker [60]. This pathway is also known as an anti-inflammatory mechanism [61]. In this study, LDH, one of the inflammatory markers, decreased after the participants consumed FRG. The abundance of Betaproteobacteria, Burkholderiales, and Sutterellaceae members increased after the intake of FRG, indicating their potential for glycolytic and anti-inflammatory functions in the host.

Our study confirmed that FRG intake alters the gut microbiota and that it has beneficial effects on host health. Galacto-oligosaccharides, prebiotics found in FRG, are food ingredients that help promote the growth of gut microbes and stimulate the activity of one or more bacterial species in the colon. It is possible that the stimulated intestinal microbial environment facilitates absorption using enzymes to metabolize the ginsenosides in FRG concentrate into small molecules [62,63]. As reported in our previous study, prebiotics are more active when taken together with probiotics. The intake of FRG with prebiotics may be another health-improving therapy [64].

Our study had several limitations. First, this was not a randomized, double-blind, and placebo-controlled study. It was a longitudinal study performed using the same technique to monitor the same subject through repeated measurements at different time points. Unlike most interventional studies, in this study, TP1 and TP3 were in the same condition, and only TP2 changed. An advantage of this approach is that we repeatedly measured the same population by changing only the time of FRG intervention. Most of the studies observed changes only before and after intake, but in this study, a wash-out period was set after intake. Although the number of participants was small, participants at TP1 and TP3 can be accurate controls. Therefore, the results of this study were directly related to FRG intake as they were based on this design. All participants displayed active participation and compliance, and there were no adverse events, deaths, or dropouts during the 9-week study period. Second, all parameters that increased or decreased at TP2 and TP3 in this study were within the normal range. Most studies have focused on patients with a specific disease, but not on improving those who do not have a specific disease or are not patients. The findings of this study revealed that the intake of FRG with probiotics may have the potential for health maintenance and disease prevention. Third, the intervention period in this study was short. The participants’ diets were artificially adjusted or controlled. However, the effect of FRG was observed for a short period, and it was confirmed that the effects were reversible when FRG intake was stopped. In addition, the associated pathways were enhanced after intake, suggesting that the direct effect is likely to increase with the duration of intake. Finally, the metabolites and ginsenosides were not measured. However, the correlation between the metabolic pathways of the gut microbiota and other variables was analyzed in depth. The findings can be used as a foundation for subsequent studies.

## 5. Conclusions

The intake of FRG for 3 weeks changed the stool consistency and frequency of bowel movements. The glucose, TG, cholesterol, LDL, creatinine, ALP, and LDH levels decreased with FRG intake, whereas the HDL level increased. We identified 20 specific bacteria s that varied abundantly after FRG intake, and they correlated with 16 metabolic pathways. This suggests that the degree of bowel movement, biochemical parameters, and metabolism can have a positive effect on individuals with a gut microbiome that overlaps with the corresponding 20 specific bacteria and the corresponding metabolic pathway. Follow-up studies are needed to observe and evaluate the effects of FRG intake on male participants and young adults. In addition, we will conduct extensive follow-up studies on health maintenance and disease prevention in healthy individuals using FRG.

## Figures and Tables

**Table 1:** Basic information of subjects in time point 1.

Variables	Value
*n* =	29
Female/Male	29/0
Age (year)	66.8 ± 7
Height (cm)	157.5 ± 4.4
Weight (kg)	58.0 ± 6.8
Waist to hip ratio (ratio)	0.9 ± 0.1
Body mass index (kg/m^2^)	23.4 ± 2.5
Systolic blood pressure (mmHg)	123.3 ± 15.5
Diastolic blood pressure (mmHg)	76.2 ± 9.5

Data are expressed as numbers or mean ± standard deviation (SD).

**Table 2:** Blood indicator values that change with FRG intake.

			TP1		TP2		TP3		*p*–Value		
			Average	SD	Average	SD	Average	SD	TP1 vs. TP2	TP2 vs. TP3	TP1 vs. TP3
Lipid panel	GLU	(mg/dL)	87	6.9	80.7	8.3	85.3	7.3	0	0	0.051
	TG	(mg/dL)	200.7	237.7	155.4	133.6	149	95.8	0.048	0.642	0.089
	CHOL	(mg/dL)	216.3	40.6	203.2	36.9	210.4	36.8	0.005	0.031	0.193
	LDL	(mg/dL)	129.6	38.1	115.4	34.4	125.7	32.2	0.002	0.007	0.37
	HDL	(mg/dL)	54.7	14.2	54	13.3	56.3	14.5	0.626	0.031	0.192
Renal function	BUN	(mg/dL)	15.5	2.9	14.9	4	14.3	3.3	0.448	0.439	0.087
	CREA	(mg/dL)	0.6	0.1	0.6	0.1	0.6	0.1	0.001	0.385	0.001
	UA	(mg/dL)	4.6	1.2	4.6	1.2	4.4	1.1	0.711	0.082	0.03
Liver function	AST	(U/L)	25.6	4.3	27.4	9.1	27	6.2	0.152	0.785	0.08
	ALT	(U/L)	19.2	6.1	21.8	9.5	20.7	8.5	0.055	0.432	0.12
	GGT	(U/L)	20	8.3	21.4	9.8	21.7	10.4	0.121	0.725	0.097
	ALP	(U/L)	77.6	19.6	72.1	18.4	74.9	18.1	0	0.002	0.013
	T-BILC	(mg/dL)	0.7	0.2	0.7	0.2	0.8	0.3	0.563	0.011	0.003
	ALB	(g/dL)	4.2	0.2	4.2	0.2	4.2	0.2	0.899	0.133	0.077
	A/G		1.2	0.1	1.4	0.2	1.3	0.1	0	0.001	0.012
	B/C		34.4	46.6	25.9	6.9	25.2	6.1	0.333	0.609	0.32
Inflammation	TP	(g/dL)	7.6	0.4	7.2	0.4	7.6	0.3	0	0	0.828
	LDH	(U/L)	187.9	35.8	177.6	31.1	175	36.5	0	0.326	0
	CRP	(mg/dL)	0.1	0.2	0.1	0.1	0.1	0.2	0.471	0.102	0.844

The mean value is shown as the average, and the deviation is shown as the standard deviation (SD). For significance values and descriptive statistics, paired *t*-test was used. Abbreviations: GLU, glucose; TG, Triglyceride; CHOL, cholesterol; LDL, low-density lipoprotein cholesterol; HDL, high-density lipoprotein cholesterol; BUN, blood urea nitrogen; CREA, creatinine; UA, uric acid; AST, aspartate aminotransferase; ALT, Alanine Aminotransferase; GGT, Gamma-glutamyl transferase; ALP, Alkaline phosphatase; T-BILC, total-bilirubin; ALB, albumin; TP, total protein; A/G ratio, albumin/Globulin ratio; B/C ratio, BUN/Creatinine ratio; TP, total protein; LDH, Lactate dehydrogenase; CRP, C-reactive protein.

## Data Availability

Data is contained within the article or supplementary material.

## Supplementary Materials

The following supporting information can be downloaded at: https://www.mdpi.com/article/10.3390/nu14091693/s1, Table S1: Ingredients of FRG; Figure S1: Test report of FRG; Table S2: Hematological Parameters by Time Point of Participants; Table S3: Analysis pf Urine Components by Time Point of Participants; Table S4: Analysis of Urine Components in TP1 of Each Participant; Table S5: Analysis of Urine Components in TP2 of Each Participant; Table S6: Analysis of Urine Components in TP3 of Each Participant.
