# Supplementary material for: Effect of Fermented Red Ginseng Concentrate Intake on Stool Characteristic, Biochemical Parameters, and Gut Microbiota in Elderly Korean Women
Source: Nutrients. 2022 Apr 19;14(9):1693. doi: 10.3390/nu14091693 (PMC9105854; doi:10.3390/nu14091693)
Supplement: Supplementary file 1 [file nutrients-14-01693-s001.zip › nutrients-1657515-supplementary-latest12052022/Figure S1.pdf]

Figure S1. Test report of FRG.

International Ginseng & Herb Research Institute

25 Insamkwangjang-ro, Geumsan-eup, Geumsan-gun, Chungnam, Republic of Korea
http://www.ginherb.re.kr/
Tel : 82-41-750-1637 FAX : 82-41-750-1629

Technovation Gin & Herb
International Ginseng & Herb Research Institute
(재)금산국제인삼약초연구소

Test Report

Client	Company name	LHK Fermentation Lab.	Issue No.	검사인증부-5828
	Address	B-616, 14, Sagimakgol-ro 45beon-gil, Jungwon-gu, Seongnam-si, Gyeonggi-do, Rep. of KOREA	Receipt No.	GHG20190924-462#1
	Tel. No./FAX No.	+82-31-731-9366 / +82-31-741-9366	Report No.	검사인증부-5828
Date of manufacture		-	Date of receipt	2019-09-24
Product Name		Fermented red ginseng	Date of Issue	2019-10-14

Test items		Results	Sum	Unit	Method	Remarks
Ginsenoside	Rg1	0.60	5.74	mg/g	Korea Food Code	-
	Re	0.89				
	Rf	0.24				
	Rg2(S)	0.14				
	Rb1	1.71				
	Rc	0.90				
	Rb2	0.69				
	Rd	0.31				
	F2	N.D				
	Rg3(S)	0.15				
	Rg3(R)	0.11				
	C.K	N.D				
	Rh2(S)	N.D				

- ▣ The above sample should be submitted and identified by the client.
- ▣ The results shown in this test refer only to the sample tested, not cover the quality of all products.
- ▣ This report can not be used for the purpose of public information, advertising and litigation without Gin&Herb's consent.
- ▣ This document cannot be reproduced without prior written approval of the client.

2019. 10. 15

Tested by

Kim Do-Yeon.

Analyst
Testing & Certification Department

Approved by

General Manager of
Testing & Certification Department

International Ginseng & Herb Research Institute
