# Supplementary material for: Effect of Fermented Red Ginseng Concentrate Intake on Stool Characteristic, Biochemical Parameters, and Gut Microbiota in Elderly Korean Women
Source: Nutrients. 2022 Apr 19;14(9):1693. doi: 10.3390/nu14091693 (PMC9105854; doi:10.3390/nu14091693)
Supplement: Supplementary file 1 [file nutrients-14-01693-s001.zip › nutrients-1657515-supplementary-latest12052022/Table S1.pdf]

Table S1. Ingredients of FRG.

Raw material name	Ratio (%)	Weight (g)
Fermented Red Ginseng Concentrate	50.000	11
Galacto oligosaccharide	9.091	2
Polydextrose	9,091	2
Vitamin B1	0.0045	0.00099
Vitamin B2	0.009	0.00198
Vitamin B6	0.009	0.00198
Water Integer	31.795	6.995
