# Supplementary material for: Effect of Fermented Red Ginseng Concentrate Intake on Stool Characteristic, Biochemical Parameters, and Gut Microbiota in Elderly Korean Women
Source: Nutrients. 2022 Apr 19;14(9):1693. doi: 10.3390/nu14091693 (PMC9105854; doi:10.3390/nu14091693)
Supplement: Supplementary file 1 [file nutrients-14-01693-s001.zip › nutrients-1657515-supplementary-latest12052022/Table S2-S6.pdf]

Table S2. Hematological Parameters by Time Point of Participants

Indicator	Unit	TP 1		TP 2		TP 3		p =		
		Average	SD	Average	SD	Average	SD	1st vs 2nd	2nd vs 3rd	1st vs 3rd
WBC	10^3 / μL	5.79	1.58	5.74	1.60	5.66	1.54	0.77	0.65	0.41
RBC	10^6 / μL	4.31	0.31	4.18	0.30	4.23	0.36	0.00	0.26	0.02
HGB	g / dL	13.30	0.62	12.99	0.64	13.20	0.83	0.00	0.05	0.32
HCT	%	39.53	2.12	38.26	2.17	38.50	2.53	0.00	0.45	0.00
MCV	fL	91.83	4.18	91.61	3.87	91.30	4.15	0.20	0.08	0.05
MCH	pg	30.90	1.46	31.12	1.52	31.31	1.45	0.01	0.01	0.00
MCHC	g / dL	33.66	0.75	33.97	0.75	34.30	0.68	0.00	0.00	0.00
PLT	10^3 / μL	248.55	54.96	243.59	49.10	245.10	55.24	0.34	0.71	0.41
RDW-SD	fL	42.49	3.26	42.39	3.38	41.79	3.61	0.59	0.01	0.01
RDW-CV	%	12.69	0.57	12.69	0.68	12.56	0.70	0.89	0.00	0.03
PDW	fL	11.37	1.65	11.13	1.61	11.16	1.62	0.17	0.78	0.24
MPV	fL	10.08	0.73	10.04	0.82	10.09	0.80	0.63	0.35	0.84
P-LCR	%	25.21	6.25	24.81	7.00	25.10	6.73	0.49	0.47	0.85
PCT	%	0.25	0.05	0.24	0.04	0.25	0.05	0.13	0.31	0.46

Abbreviation: WBC, white blood cells; RBC. red blood cells; HGB, hemoglobin; HCT, hematocrit; MCV, mean corpuscular volume; MCH, mean corpuscular hemoglobin; MCHC, mean corpuscular hemoglobin concentration; PLT, platelet; RDW-SD, red cell distribution width; RDW-CV, red cell distribution width - coefficient of variation; PDW, Platelet distribution width; MPV, mean platelet volume; P-LCR, Platelet-large cell ratio; PCT, Procalcitonin.
For statistics, paired t-test was performed.

Table S3. Analysis pf Urine Components by Time Point of Participants

	TP 1		TP 2		TP 3		*p* =		
	Average	SD	Average	SD	Average	SD	1st vs 2nd	2nd vs 3rd	1st vs 3rd
BLD	0.90	1.65	1.21	1.94	1.45	2.13	0.13	0.40	0.04
BIL	0.10	0.55	0.10	0.55	0.24	0.90	1.00	0.50	0.33
URO	1.00	0.00	1.00	0.00	1.00	0.00	1.00	1.00	1.00
KET	0.48	0.97	1.00	1.23	1.03	1.10	0.07	0.90	0.02
pH	6.33	0.62	6.26	0.50	6.41	0.68	0.65	0.37	0.55
S.G	1.02	0.00	1.02	0.01	1.02	0.01	0.89	0.43	0.15
LFU	1.59	1.97	1.14	1.63	1.59	2.11	0.24	0.26	1.00

Abbreviation: BLD, blood; BIL, bilirubin; URO, urobilinogen; KET, ketones body; pH, potential of hydrogen; S.G, specific gravity; LFU, Leukocyte esterase urine.
For statistics, paired t-test was performed.
Numerical Conversion: 0, negative; 1, normal; 2, semi-positive; 3, 1-positive; 4, 2-positive; 5, 3-positive.
Specific gravity and pH are integer values when measured without following numerical conversion.

Table S4. Analysis of Urine Components in TP 1 of Each Participant

no.	TP 1						
	BLD	BIL	URO	KET	pH	S.G	LFU
1	3	0	1	0	6	1.025	5
2	0	0	1	0	6	1.025	0
3	0	0	1	0	6	1.03	5
4	0	0	1	0	6.5	1.015	0
5	3	0	1	0	6	1.025	3
6	0	0	1	0	6	1.015	0
7	5	0	1	2	6	1.025	0
8	0	3	1	0	8	1.015	0
9	0	0	1	0	6	1.015	0
10	0	0	1	0	6	1.015	4
11	0	0	1	0	6	1.02	3
12	0	0	1	0	6	1.025	0
13	0	0	1	0	6.5	1.02	0
14	0	0	1	2	6	1.03	5
15	5	0	1	0	6	1.02	0
16	3	0	1	2	6.5	1.015	0
17	0	0	1	0	6	1.015	0
18	0	0	1	0	8.5	1.015	3
19	0	0	1	0	6	1.025	3
20	0	0	1	2	7	1.02	3
21	0	0	1	0	6.5	1.01	0
22	0	0	1	0	6	1.025	5
23	4	0	1	0	6	1.015	3
24	0	0	1	3	6	1.025	0
25	3	0	1	0	6	1.02	0
26	0	0	1	3	7	1.02	0
27	0	0	1	0	6	1.02	4
28	0	0	1	0	6	1.02	0
29	0	0	1	0	7	1.02	0

Abbreviation: BLD, blood; BIL, bilirubin; URO, urobilinogen; KET, ketones body; pH, potential of hydrogen; S.G, specific gravity; LFU, Leukocyte esterase urine

Numerical Conversion: 0, negative; 1, normal; 2, semi-positive; 3, 1-positive; 4, 2-positive; 5, 3-positive. Specific gravity and pH are integer values when measured without following numerical conversion.

Table S5. Analysis of Urine Components in TP 2 of Each Participant

no.	TP 2						
	BLD	BIL	URO	KET	pH	S.G	LFU
1	5	0	1	3	6	1.025	4
2	0	0	1	2	6	1.025	0
3	0	0	1	0	6	1.02	5
4	0	0	1	3	6	1.02	0
5	3	0	1	0	6	1.025	0
6	2	0	1	0	6.5	1.01	0
7	5	3	1	3	6	1.025	0
8	3	0	1	0	6	1.025	3
9	0	0	1	2	6	1.02	0
10	0	0	1	0	6	1.01	3
11	0	0	1	0	7	1.015	0
12	2	0	1	0	6	1.025	0
13	0	0	1	3	6	1.02	0
14	0	0	1	2	7.5	1.015	0
15	5	0	1	0	6	1.025	0
16	0	0	1	0	7.5	1.015	0
17	0	0	1	0	7.5	1.015	0
18	0	0	1	0	6	1.025	4
19	0	0	1	0	6	1.025	0
20	0	0	1	2	7	1.02	3
21	0	0	1	2	6	1.025	2
22	0	0	1	0	6	1.025	0
23	5	0	1	2	6	1.025	0
24	0	0	1	0	6	1.02	0
25	5	0	1	3	6	1.025	3
26	0	0	1	2	6	1.02	0
27	0	0	1	0	6	1.01	3
28	0	0	1	0	6	1.02	3
29	0	0	1	0	6.5	1.015	0

Abbreviation: BLD, blood; BIL, bilirubin; URO, urobilinogen; KET, ketones body; pH, potential of hydrogen; S.G, specific gravity; LFU, Leukocyte esterase urine

Numerical Conversion: 0, negative; 1, normal; 2, semi-positive; 3, 1-positive; 4, 2-positive; 5, 3-positive. Specific gravity and pH are integer values when measured without following numerical conversion.

Table S6. Analysis of Urine Components in TP 3 of Each Participant

no.	TP 3						
	BLD	BIL	URO	KET	pH	S.G	LFU
1	5	0	1	0	6	1.025	5
2	2	0	1	2	6	1.02	5
3	0	0	1	0	6	1.03	5
4	0	0	1	2	7.5	1.015	0
5	3	4	1	0	6	1.03	0
6	5	0	1	2	6	1.02	3
7	5	0	1	2	6	1.025	0
8	0	3	1	2	6.5	1.02	0
9	0	0	1	0	7	1.015	0
10	0	0	1	2	6.5	1.02	3
11	0	0	1	0	6	1.025	0
12	5	0	1	0	7	1.025	0
13	0	0	1	0	8.5	1.015	0
14	0	0	1	2	6	1.025	0
15	5	0	1	3	6	1.02	3
16	5	0	1	0	6	1.025	5
17	0	0	1	0	7	1.015	0
18	0	0	1	0	7.5	1.02	4
19	0	0	1	2	6	1.025	0
20	0	0	1	2	6.5	1.02	3
21	0	0	1	2	8	1.01	0
22	0	0	1	0	6	1.025	5
23	5	0	1	0	6	1.025	0
24	0	0	1	3	6	1.025	0
25	2	0	1	2	6	1.02	0
26	0	0	1	2	6	1.025	0
27	0	0	1	0	6	1.02	5
28	0	0	1	0	6	1.01	0
29	0	0	1	0	6	1.025	0

Abbreviation: BLD, blood; BIL, bilirubin; URO, urobilinogen; KET, ketones body; pH, potential of hydrogen; S.G, specific gravity; LFU, Leukocyte esterase urine

Numerical Conversion: 0, negative; 1, normal; 2, semi-positive; 3, 1-positive; 4, 2-positive; 5, 3-positive. Specific gravity and pH are integer values when measured without following numerical conversion.
